# Dr. Amos L. Sweet

**Published:** 1917-02

**Authors:** 


**Dr. Amos L. Sweet,** N. Y. University 1866, died at his home in Geneva, Jan. 2, aged 70.

